# Genome sequence of *Burkholderia mimosarum* strain LMG 23256^T^, a *Mimosa pigra* microsymbiont from Anso, Taiwan

**DOI:** 10.4056/sigs.4848627

**Published:** 2013-12-31

**Authors:** Anne Willems, Rui Tian, Lambert Bräu, Lynne Goodwin, James Han, Konstantinos Liolios, Marcel Huntemann, Amrita Pati, Tanja Woyke, Konstantinos Mavrommatis, Victor Markowitz, Natalia Ivanova, Nikos Kyrpides, Wayne Reeve

**Affiliations:** 1 Laboratory of Microbiology, Department of Biochemistry and Microbiology, Faculty of Sciences, Ghent University, Begium; 2 Centre for Rhizobium Studies, Murdoch University, Western Australia, Australia; 3 School of Life and Environmental Sciences, Deakin University, Victoria, Australia; 4 Los Alamos National Laboratory, Bioscience Division, Los Alamos, New Mexico, USA; 5 DOE Joint Genome Institute, Walnut Creek, California, USA; 6 Biological Data Management and Technology Center, Lawrence Berkeley National Laboratory, Berkeley, California, USA

**Keywords:** root-nodule bacteria, nitrogen fixation, rhizobia, *Betaproteobacteria*

## Abstract

*Burkholderia mimosarum* strain LMG 23256^T^ is an aerobic, motile, Gram-negative, non-spore-forming rod that can exist as a soil saprophyte or as a legume microsymbiont of *Mimosa pigra* (giant sensitive plant). LMG 23256^T^ was isolated from a nodule recovered from the roots of the *M. pigra* growing in Anso, Taiwan. LMG 23256^T^ is highly effective at fixing nitrogen with *M. pigra*. Here we describe the features of *B. mimosarum* strain LMG 23256^T^, together with genome sequence information and its annotation. The 8,410,967 bp high-quality-draft genome is arranged into 268 scaffolds of 270 contigs containing 7,800 protein-coding genes and 85 RNA-only encoding genes, and is one of 100 rhizobial genomes sequenced as part of the DOE Joint Genome Institute 2010 Genomic Encyclopedia for Bacteria and Archaea-Root Nodule Bacteria (GEBA-RNB) project.

## Introduction

Members of the versatile genus *Burkholderia* occupy a wide range of ecological niches and are found in soil, hospital environments, associated with plants either as epiphytes, endophytes or as pathogens and some are endosymbionts in phytopathogenic fungi or plant-associated insects [1]. As several *Burkholderia* strains are known to exert plant-beneficial and biocontrol effects, and also contribute to adaptation to environmental stresses, there is increased interest in the use of *Burkholderia* in agriculture [1,2].

In addition to the different groups of rhizobia from the *Alphaproteobacteria*, a number of *Betaproteobacteria* belonging to *Burkholderia* and *Cupriavidus* are now also known to be present in legume nodules; they are sometimes referred to as betarhizobia [3-5]. Several *Burkholderia* species have been described from root nodules of different *Mimosa* species: *B. caribensis* from *M. pudica* and *M. diplotricha* [4,6], *B. mimosarum* from *M. pigra* and *M. scabrella* [7], *B. nodosa* from *M. bimucronata* and *M. scabrella* [8], *B. phymatum* from *M. invisa* and *Machaerium lunatum* [6,9] and *B. sabiae* from *M. caesalpiniifolia* [10]. Moreover, several *Burkholderia* strains have been shown to enter into effective symbiosis with their host [11].

*B. mimosarum* was described for a collection of isolates obtained from *M. pigra* in Taiwan, Venezuela and Brazil and one strain from *M. scabrella* in Brazil [7]. Since its first description, *B. mimosarum* has also been isolated from *M. pigra* nodules in China and Australia [12,13], from *M. diplotricha* in Papua New Guinea [14] and *M. pudica* in French Guiana [15]. *M. pigra*, as well as *M. pudica* and *M. diplotricha*, are notoriously invasive species [16]. *M. pudica* (sensitive plant) is a small South American shrub that has become a pan-tropical weed, while *M. pigra* (giant sensitive plant, black mimosa, prickly wood weed, catclaw mimosa) is a shrub that thrives in floodplains, swamps and river banks, where it creates dense spiny thickets [17]. *M. diplotricha* (creeping sensitive plant, nila grass, giant sensitive plant) is a climbing shrub that scrambles up other plants, quickly producing dense growth [18]. The success of these invasive weeds may in part be due to their highly effective symbiotic associations.

*B. mimosarum* LMG 23256^T^ (=BCRC 17516, CCUG 54296, NBRC 106338, PAS44) originates from nodules of *M. pigra* in Taiwan. This legume weed is predominantly nodulated by *B. mimosarum* in Taiwan. Other Taiwanese *Mimosa* species are nodulated mainly by *Cupriavidus taiwanensis* and it has therefore been suggested that the *Burkholderia* strains were introduced to Taiwan, along with the invasive *M. pigra* from its native South America, where *Burkholderia* strains have been isolated more frequently from *Mimosa* sp. than *C. taiwanesis* [7,19].

Here we present a summary classification and a set of features for *B. mimosarum* strain LMG 23256^T^ (Table 1), together with the description of the complete genome sequence and its annotation.

**Table 1:** Classification and general features of *Burkholderia mimosarum* strain LMG 23256^T^ according to the MIGS recommendations [20]

**MIGS ID**	**Property**	**Term**	**Evidence code**
	Current classification	Domain *Bacteria*	TAS [21]
		Phylum *Proteobacteria*	TAS [22]
		Class *Betaproteobacteria*	TAS [23,24]
		Order *Burkholderiales*	TAS [24,25]
		Family *Burkholderiaceae*	TAS [24,26]
		Genus *Burkholderia*	TAS [27-29]
		Species *Burkholderia mimosarum*	TAS [7]
		Strain LMG 23256^T^	
	Gram stain	Negative	IDA
	Cell shape	Rod	IDA
	Motility	Motile	IDA
	Sporulation	Non-sporulating	NAS
	Temperature range	Mesophile	NAS
	Optimum temperature	28°C	NAS
	Salinity	Non-halophile	NAS
MIGS-22	Oxygen requirement	Aerobic	TAS [19]
	Carbon source	Varied	NAS
	Energy source	Chemoorganotroph	NAS
MIGS-6	Habitat	Soil, root nodule, on host	TAS [19]
MIGS-15	Biotic relationship	Free living, symbiotic	TAS [19]
MIGS-14	Pathogenicity	Non-pathogenic	NAS
	Biosafety level	1	TAS [30]*
	Isolation	Root nodule of *Mimosa pigra*	TAS [19]
MIGS-4	Geographic location	Anso, Taiwan	TAS [19]
MIGS-5	Soil collection date	Not recorded	IDA
MIGS-4.1	Longitude	120.87222	IDA
MIGS-4.2	Latitude	22.28889	
MIGS-4.3	Depth	Not recorded	IDA
MIGS-4.4	Altitude	Not recorded	IDA

*Strain catalogue BCCM/LMG http://bccm.belspo.be/db/lmg_search_form.php

Evidence codes – IDA: Inferred from Direct Assay; TAS: Traceable Author Statement (i.e., a direct report exists in the literature); NAS: Non-traceable Author Statement (i.e., not directly observed for the living, isolated sample, but based on a generally accepted property for the species, or anecdotal evidence). These evidence codes are from the Gene Ontology project [31].

## Classification and features

**Figure 1:**
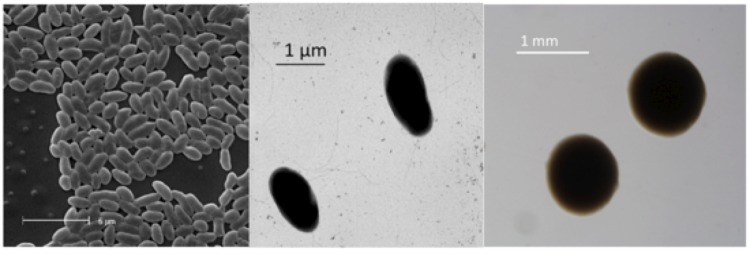
Images of *Burkholderia mimosarum* strain LMG 23256^T^ using scanning (Left) and transmission (Center) electron microscopy and the appearance of colony morphology on a solid medium (Right).

**Figure 2:**
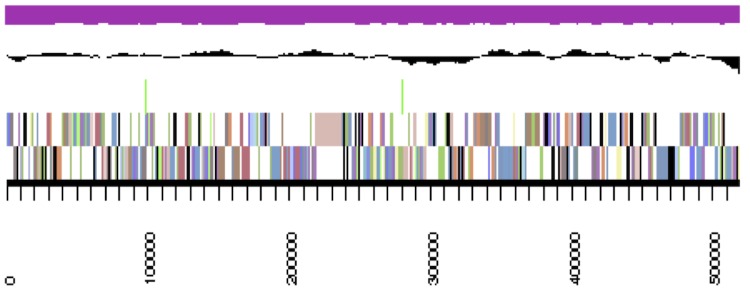
Phylogenetic tree showing the relationship of *Burkholderia mimosarum* strain LMG 23256^T^ (shown in bold print) to other members of the order *Burkholderiales* based on aligned sequences of the 16S rRNA gene (1,242 bp internal region). All sites were informative and there were no gap-containing sites. Phylogenetic analyses were performed using MEGA, version 5 [35]. The tree was built using the Maximum-Likelihood method with the General Time Reversible model [36]. Bootstrap analysis [37] with 500 replicates was performed to assess the support of the clusters. Type strains are indicated with a superscript T. Brackets after the strain name contain a DNA database accession number and/or a GOLD ID (beginning with the prefix G) for a sequencing project registered in GOLD [38]. Published genomes are indicated with an asterisk.

*B. mimosarum* strain LMG 23256^T^ is a non-sporulating, non-encapsulated, Gram-negative rod within the order *Burkholderiales* of the class *Betaproteobacteria*. The rod-shaped form varies in size; it is approximately 1.0 μm in width and 2.0 μm in length (Figure 1, Left and Figure 1, Center). It is fast-growing, forming colonies within 3-4 days when grown on half strength Lupin Agar (½LA) [32], tryptone-yeast extract agar (TY) [33] or a modified yeast-mannitol agar (YMA) [34] at 28°C. Colonies on ½LA are white-opaque, slightly domed and moderately mucoid with smooth margins (Figure 1, Right). Minimum Information about the Genome Sequence (MIGS) is provided in Table 1. Figure 2 shows the phylogenetic neighborhood of *B. mimosarum* strain LMG 23256^T^ in a 16S rRNA sequence based tree. This strain shares 99% (1,121/1,124 bp) and 98% (1,101/1,125 bp) sequence identity to the 16S rRNA of the fully sequenced strain *B. mimosarum* STM3621 (Gi08839) and to *B. nodosa* Br3461^T^, respectively.

### Symbiotaxonomy

*B. mimosarum* LMG 23256^T^ was isolated from *M. pigra* growing in Anso, Taiwan and was able to nodulate its original host with high efficiency [19], as well as *M. pucida* and *M. diplotricha* [14]. LMG 23256^T^ was shown to outcompete other rhizobia to the point of exclusion for the nodulation of the invasive *M. pigra*, *M. pudica* and *M. diplotricha* under flooded conditions. This predominance was negatively affected by increased nitrate levels in the soil, which thus seems to be a factor affecting rhizobial competition [14].

With regard to other plant growth promoting properties, LMG 23256^T^ displayed no antifungal activity against *Fusarium oxysporum* f. sp. *phaseoli*, did not solubilize calcium-, iron- or aluminum phosphates nor reduce acetylene (ARA) on the N-free media containing fructose, lactate or mannitol as sole carbon source [39].

## Genome sequencing and annotation

### Genome project history

This organism was selected for sequencing on the basis of its environmental and agricultural relevance to issues in global carbon cycling, alternative energy production, and biogeochemical importance, and is part of the Community Sequencing Program at the U.S. Department of Energy, Joint Genome Institute (JGI) for projects of relevance to agency missions. The genome project is deposited in the Genomes OnLine Database [38] and an improved-high-quality-draft genome sequence in IMG. Sequencing, finishing and annotation were performed by the JGI. A summary of the project information is shown in Table 2.

**Table 2:** Genome sequencing project information for *Burkholderia mimosarum* LMG 23256^T^.

**MIGS ID**	**Property**	**Term**
MIGS-31	Finishing quality	Improved high-quality draft
MIGS-28	Libraries used	One Illumina fragment library
MIGS-29	Sequencing platforms	Illumina HiSeq 2000
MIGS-31.2	Sequencing coverage	Illumina: 240×
MIGS-30	Assemblers	Velvet version 1.1.04; Allpaths-LG version r39750
MIGS-32	Gene calling methods	Prodigal 1.4
	GOLD ID	Gi08823
	NCBI project ID	163559
	Database: IMG	2513237083
	Project relevance	Symbiotic N_2_ fixation, agriculture

### Growth conditions and DNA isolation

*B. mimosarum* strain LMG 23256^T^ was cultured to mid logarithmic phase in 60 ml of TY rich medium on a gyratory shaker at 28°C [40]. DNA was isolated from the cells using a CTAB (Cetyl trimethyl ammonium bromide) bacterial genomic DNA isolation method (http://my.jgi.doe.gov/general/index.html).

### Genome sequencing and assembly

The genome of *B. mimosarum* strain LMG 23256^T^ was sequenced at the Joint Genome Institute (JGI) using Illumina technology [41]. An Illumina standard shotgun library was constructed and sequenced using the Illumina HiSeq 2000 platform, which generated 14,635,038 reads totaling 2,014 Mbp.

All general aspects of library construction and sequencing performed at the JGI can be found at http://my.jgi.doe.gov/general/index.html. All raw Illumina sequence data was passed through DUK, a filtering program developed at JGI, which removes known Illumina sequencing and library preparation artifacts (Mingkun, L., Copeland, A. and Han, J., unpublished). The following steps were then performed for assembly: (1) filtered Illumina reads were assembled using Velvet [42] (version 1.1.04), (2) 1–3 Kbp simulated paired end reads were created from Velvet contigs using wgsim [43], (3) Illumina reads were assembled with simulated read pairs using Allpaths–LG [44] (version r39750). Parameters for assembly steps were:

1. Velvet (--v --s 51 --e 71 --i 2 --t 1 --f "-shortPaired -fastq $FASTQ" --o "-ins_length 250 -min_contig_lgth 500") 10)
2. wgsim (-e 0 -1 76 -2 76 -r 0 -R 0 -X 0)
3. Allpaths–LG (PrepareAllpathsInputs:PHRED64=1 PLOIDY=1 FRAGCOVERAGE=125 JUMPCOVERAGE=25 LONGJUMPCOV=50, RunAllpath-sLG: THREADS=8 RUN=stdshredpairs TARGETS=standard VAPIWARNONLY=True OVERWRITE=True).

The final draft assembly contained 270 contigs in 268 scaffolds. The total size of the genome is 8.4 Mbp and the final assembly is based on 2,014 Mbp of Illumina data, which provides an average 240× coverage of the genome.

### Genome annotation

Genes were identified using Prodigal [45] as part of the DOE-JGI annotation pipeline [46]. The predicted CDSs were translated and used to search the National Center for Biotechnology Information (NCBI) nonredundant database, UniProt, TIGRFam, Pfam, PRIAM, KEGG, COG, and InterPro databases. The tRNAScanSE tool [47] was used to find tRNA genes, whereas ribosomal RNA genes were found by searches against models of the ribosomal RNA genes built from SILVA [48]. Other non–coding RNAs such as the RNA components of the protein secretion complex and the RNase P were identified by searching the genome for the corresponding Rfam profiles using INFERNAL [49]. Additional gene prediction analysis and manual functional annotation was performed within the Integrated Microbial Genomes (IMG-ER) platform [50].

## Genome properties

The genome is 8,410,967 nucleotides 63.89% GC content (Table 3) and comprised of 268 scaffolds (the four largest scaffolds are shown in Figures 3a, 3b, 3c and Figure 3d) of 270 contigs. From a total of 7,885 genes, 7,800 were protein encoding and 85 RNA only encoding genes. The majority of genes (75.13%) were assigned a putative function whilst the remaining genes were annotated as hypothetical. The distribution of genes into COGs functional categories is presented in Table 4.

**Table 3:** Genome Statistics for *B. mimosarum* strain LMG 23256^T^

**Attribute**	**Value**	**% of Total**
Genome size (bp)	8,410,967	100.00
DNA coding region (bp)	7,084,175	84.23
DNA G+C content (bp)	5,373,761	63.89
Number of scaffolds	268	
Number of contigs	270	
Total gene	7,885	100.00
RNA genes	85	1.08
rRNA operons*	1	0.01
Protein-coding genes	7,800	98.92
Genes with function prediction	5,924	75.13
Genes assigned to COGs	5,870	74.45
Genes assigned Pfam domains	6,242	79.16
Genes with signal peptides	673	8.54
Genes with transmembrane helices	1,680	21.31
CRISPR repeats	0	

*5 copies of 5S, 1 copy of 16S and 2 copies of 23S rRNA.

**Figure 3a:**
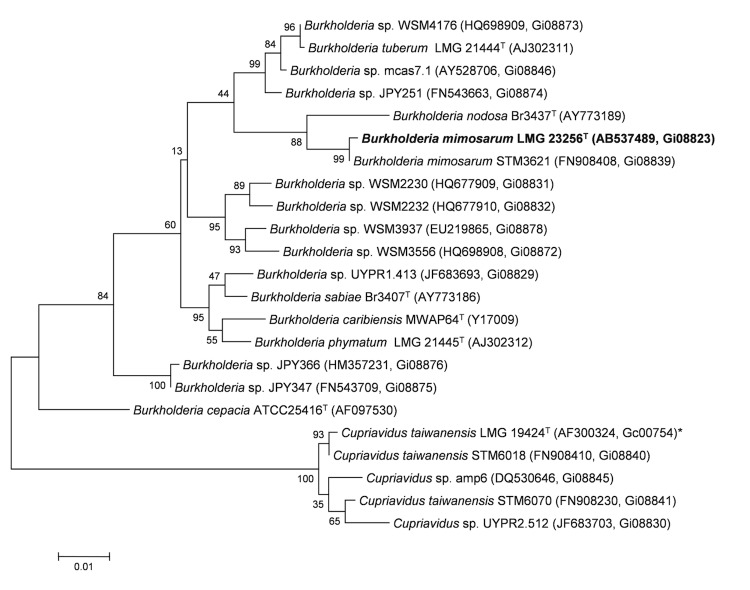
Graphical map of LMG 23256_A19UDRAFT_scaffold_0.1 of the *B. mimosarum* strain LMG 23256^T^ genome. From bottom to the top of each scaffold: Genes on forward strand (color by COG categories as denoted by the IMG platform), Genes on reverse strand (color by COG categories), RNA genes (tRNAs green, sRNAs red, other RNAs black), GC content, GC skew.

**Figure 3b:**
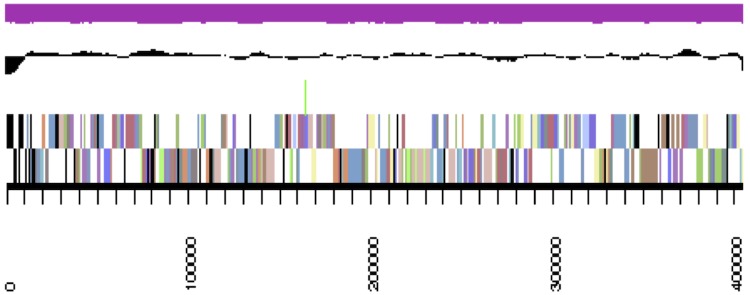
Graphical map of LMG 23256_A19UDRAFT_scaffold_1.2 of the *B. mimosarum* strain LMG 23256^T^ genome. From bottom to the top of each scaffold: Genes on forward strand (color by COG categories as denoted by the IMG platform), Genes on reverse strand (color by COG categories), RNA genes (tRNAs green, sRNAs red, other RNAs black), GC content, GC skew.

**Figure 3c:**
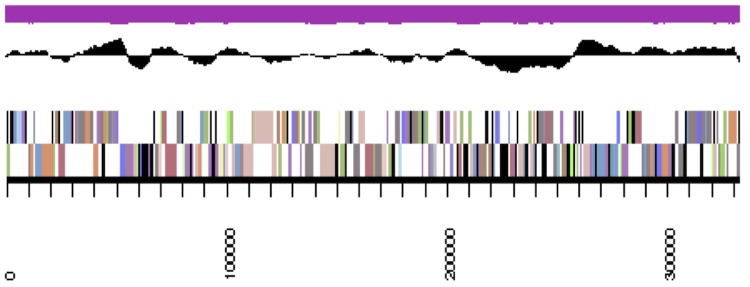
Graphical map of LMG 23256_A19UDRAFT_scaffold_2.3 of the *B. mimosarum* strain LMG 23256^T^ genome. From bottom to the top of each scaffold: Genes on forward strand (color by COG categories as denoted by the IMG platform), Genes on reverse strand (color by COG categories), RNA genes (tRNAs green, sRNAs red, other RNAs black), GC content, GC skew.

**Figure 3d:**
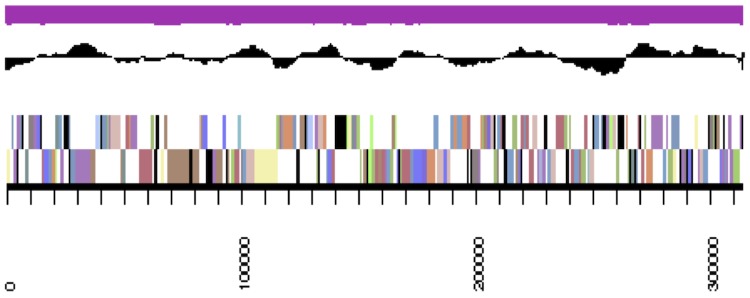
Graphical map of LMG 23256_A19UDRAFT_scaffold_3.4 of the *B. mimosarum* strain LMG 23256^T^ genome. From bottom to the top of each scaffold: Genes on forward strand (color by COG categories as denoted by the IMG platform), Genes on reverse strand (color by COG categories), RNA genes (tRNAs green, sRNAs red, other RNAs black), GC content, GC skew.

**Table 4:** Number of protein coding genes of *B. mimosarum* strain LMG 23256^T^ associated with the general COG functional categories.

Code	Value	%age	Description
J	191	2.89	Translation, ribosomal structure and biogenesis
A	6	0.09	RNA processing and modification
K	588	8.89	Transcription
L	415	6.28	Replication, recombination and repair
B	2	0.03	Chromatin structure and dynamics
D	50	0.76	Cell cycle control, mitosis and meiosis
Y	0	0.00	Nuclear structure
V	71	1.07	Defense mechanisms
T	376	5.69	Signal transduction mechanisms
M	414	6.26	Cell wall/membrane biogenesis
N	146	2.21	Cell motility
Z	0	0.00	Cytoskeleton
W	0	0.00	Extracellular structures
U	161	2.43	Intracellular trafficking and secretion
O	208	3.15	Posttranslational modification, protein turnover, chaperones
C	489	7.39	Energy production conversion
G	435	6.58	Carbohydrate transport and metabolism
E	623	9.42	Amino acid transport metabolism
F	98	1.48	Nucleotide transport and metabolism
H	226	3.42	Coenzyme transport and metabolism
I	316	4.78	Lipid transport and metabolism
P	293	4.43	Inorganic ion transport and metabolism
Q	231	3.49	Secondary metabolite biosynthesis, transport and catabolism
R	745	11.27	General function prediction only
S	529	8.00	Function unknown
-	2,015	25.55	Not in COGS
	6,612	-	Total
